# Comparison of inbred mouse substrains reveals segregation of maladaptive fear phenotypes

**DOI:** 10.3389/fnbeh.2014.00282

**Published:** 2014-08-20

**Authors:** Stephanie J. Temme, Ryan Z. Bell, Reciton Pahumi, Geoffrey G. Murphy

**Affiliations:** ^1^Neuroscience Graduate Program, University of MichiganAnn Arbor, MI, USA; ^2^Molecular and Behavioral Neuroscience Institute, University of MichiganAnn Arbor, MI, USA; ^3^Department of Molecular and Integrative Physiology, University of MichiganAnn Arbor, MI, USA

**Keywords:** maladaptive fear, inbred mouse strains, conditioned fear, extinction, generalization, context discrimination, 129S1, 129S6

## Abstract

Maladaptive fear, such as fear that is persistent or easily generalized to a nonthreatening stimuli, is associated with anxiety-related disorders in humans. In the laboratory, maladaptive fear can be modeled in rodents using Pavlovian fear conditioning. Recently, an inbred mouse strain known as 129S1/SvImJ, or 129S1 has been reported as exhibiting impairments in fear extinction and enhanced fear generalization. With a long-term goal of identifying segregating genetic markers of maladaptive fear, we used Pavlovian fear conditioning to characterize a closely related substrain designated as 129S6/SvEvTac, or 129S6. Here we report that, like 129S1 animals, 129S6 mice exhibit appropriate levels of fear upon conditioning, but are unable to extinguish fear memories once they are consolidated. Importantly, the maladaptive fear phenotype in this inbred stain can be segregated by sub-strain when probed using conditioning protocols designed to assess generalized fear. We find that unlike the 129S1 substrain, mice from the 129S6 sub-strain do not generalize conditioned fear to previously novel contexts and can learn to discriminate between two similar contexts when trained using a discrimination protocol. These results suggest that at least two forms of maladaptive fear (deficits in fear extinction and fear generalization) can be can be functionally segregated, further suggesting that the underlying neurobiology is heritable. Given the observation that two closely related sub-strains can exhibit different constellations of maladaptive fear suggests that these findings could be exploited to facilitate the identification of candidate genes for anxiety-related disorders.

## Introduction

Fear can be both adaptive and maladaptive (Bracha, 2006). While adaptive fears helps protect against injury or death, maladaptive fears often result in anxiety-related and trauma-related disorders, such as post-traumatic stress disorder (PTSD; Bracha, 2006).

In a laboratory setting, fear is often studied through Pavlovian fear conditioning, in which a neutral conditioned stimulus (CS), such as a context or a tone, is paired with an aversive unconditioned stimulus (US), such as a footshock (Maren, 2005; Milad et al., 2006; Orsini and Maren, 2012). Following paired presentations of the CS and US, the previously neutral CS alone is enough to produce a fear response, often quantified as freezing, or inactivity of the animal except for that required for respiration. Freezing to a CS can be subsequently reduced through Pavlovian fear extinction, in which the CS is presented multiple times in the absence of the US. It is generally accepted that the extinction of conditioned fear represents the learning of a new association and not the erasure of the original fear memory (Rescorla and Heth, 1975; Bouton and King, 1983; Rescorla, 2001). Under normal conditions, fear extinction learning is viewed as adaptive (Milad et al., 2006; Rauch et al., 2006). Conversely, deficits in fear extinction are considered to be maladaptive and may be related to anxiety-related and trauma-related disorders (Milad et al., 2006; Rauch et al., 2006).

In an attempt to identify genetic components that may modulate maladaptive fear learning and anxiety-related disorders, previous studies have compared various inbred mouse lines for abnormalities in Pavlovian fear conditioning and extinction (Trullas and Skolnick, 1993; Crawley et al., 1997; Bolivar et al., 2001; Holmes et al., 2002; Balogh and Wehner, 2003; Bothe et al., 2004; Hefner et al., 2008; Camp et al., 2009; Wilkinson et al., 2013). In these studies, the C57B6 strain is considered to exhibit “normal” fear learning and extinction. Studies examining the 129 inbred strain have found that the 129S1 substrain acquires and consolidates fear memories (Bolivar et al., 2001; Hefner et al., 2008), but is unable to extinguish fear of a previously trained CS when compared to the C57B6 inbred strain (Hefner et al., 2008). Further studies of the 129S1 substrain found that, once conditioned to fear a context, 129S1 mice over-generalized their fear to non-conditioned contexts relative to C57B6 mice (Camp et al., 2012). Based on these studies, genetic comparison of 129S1 and C57B6, using techniques such as DNA microarrays could result in a list of genes that mediate maladaptive fear. However, significant genetic diversity between the 129S1 and C57B6 inbred strains make the identification of these genes difficult. If differences in maladaptive fear could be found between two more genetically similar substrains, such as 129S1 and 129S6 mice (Simpson et al., 1997), the selection of candidate genes contributing to the 129S1 phenotype could be significantly facilitated. While a previous study has demonstrated that additional 129 substrains exhibit similar deficits in extinction (Camp et al., 2009), little else is known about the behavior and neurobiological differences between 129 substrains. In particular, it remains unknown whether the commonly used 129S6 substrain exhibits similar maladaptive fear. Based on the development of the 129 lineages and substrains, the genetic variation between the 129S1 and 129S6 should be significantly reduced compared to C57B6 and other inbred mouse strains (Simpson et al., 1997; Threadgill et al., 1997).

To determine whether 129S6 mice exhibit maladaptive fear, we fear conditioned 129S6 mice to a context or tone and compared their levels of fear consolidation, fear extinction, and fear generalization to C57B6 mice. In addition, we directly compared 129S1 and 129S6 in context discrimination and generalization.

The results from these studies suggest that the 129S6 and 129S1 substrains share some aspects of maladaptive fear and not others. While the 129S6 substrain conditions to fear normally, mice in this substrain are unable to extinguish this conditioned fear, similar to the previous published 129S1 mice. However, the 129S6 did not exhibit aberrant fear generalization or context discrimination while 129S1 mice do, illustrating key differences in maladaptive fear between the two strains. Based on these studies, we conclude that deficits in fear extinction and fear generalization/discrimination likely represent independent forms of maladaptive fear. In this way, further comparison of 129S1 and 129S6 mice may help shed light on the genetic underpinnings of these psychiatric disease states. Additionally, this divergence in forms of maladaptive fear may be important for understanding the development and maintenance of trauma and anxiety-related disorders.

## Materials and methods

### Mice

All mice were either obtained from their respective vendors or bred within our colony using naïve mice from the same vendors. The 129SvEvS6/Tac and C57BL/6NTac mice were obtained from Taconic Farms (Hudson, NY) and 129S1/SvImJ mice were obtained from Jackson Laboratories (Bar Harbor ME) and are referred to hereafter as 129S6, C57B6, and 129S1, respectively.

Studies were conducted using mice aged 3–6 months at the time of testing with approximately equal numbers of males and females. All mice were housed by sex in groups of 2–5. Mice were maintained in micro-isolation cages with a 14-h/10-h light/dark cycle for a minimum of 1 week prior to behavioral studies. The average ambient temperature was 22°C and mice were provided with ad libitum food and water. All experiments were conducted according to the National Institute of Health guidelines for animal care and were approved by the University Committee on the Use and Care of Animals of the University of Michigan.

### Behavioral procedures

#### Conditioning apparatus and contexts

All experiments were conducted in fear conditioning chambers with clear acrylic backs and doors, aluminum sides, stainless steel grid floors spaced 1/8 inches, and stainless steel drop pans (Med Associates). Shocks were administered through the grid via solid-state shock scramblers and electronic constant-current shock sources controlled by a desktop PC running Actimetrics Freezeframe software (Wilmette, IL). The same computer and software were used to record behavior which was digitized using individual cameras mounted above each chamber. Individual chamber details and room lighting were altered to create three experimental contexts termed “same, A”, “similar, B”, and “different, C”. Context A was created using the basic conditioning chamber described above and white room lights set at 150 watts. Chambers and floor pans were cleaned with 70% ethanol to provide a distinct background odor. Context B was identical to context A with the addition of rubber speckled floor coverings over smooth acrylic coverings to hide grid floors. Context C included smooth opaque white acrylic coverings over the floor and walls which produced the appearance of a semicircular chamber. The chamber and floor pans in context C were cleaned with 2% acetic acid and red room lights at 60 watts were used. For experimental sessions using contexts A or B, mice were transferred to a holding room prior to the beginning of the session. For context C, mice were transferred directly from their housing room to the experimental set-up at the start of the session. In all contexts, freezing was defined as a lack of motion, except that required for respiration, for 1 s or more and was calculated using a sensitive global motion-detection algorithm (FreezeFrame and FreezeView software; Actimetrics; Wilmette, IL).

#### Protocols

##### Context protocols

During context conditioning, mice were trained for 3 days using one training session per day. Throughout training, mice were placed in context A in individual conditioning chambers. Each training session was composed of 3 min of baseline activity in context A followed by three unsignaled foot-shocks (2 s) with 30 s postshock intervals. Mice remained in the conditioning chamber for 30 s following the last footshock. Based on previous literature (Smith et al., 2007; Matynia et al., 2008) and our prior work, strain specific shock intensities were used (0.5 mA for 129S1 and 129S6 mice and 0.75 mA for C57B6 mice) to elicit similar levels of freezing while preventing over/undertraining. After training, mice were tested in one of three contexts: same, A; similar, B; and different, C. All context tests consisted of 5 min of context exposure.

For experiments that examined context extinction, mice were counterbalanced for their test context and subsequently divided into two groups: extinction and no extinction. Twenty-four hours after testing, mice in the extinction group were extinguished to context A using 60 min of context exposure divided evenly across 2 days. Mice in the no extinction group remained in their home-cage as a retention control group. Twenty-four hours after extinction, both extinction and no extinction mice were tested for their fear in context A using 5 min of context exposure.

During context discrimination, mice were trained to discriminate through exposure to both context A and context B each day for 9 days, separated by a minimum of 6 h. The order of exposure to contexts A and B was alternated each day. In context A, mice were trained each day using 3 min of context exposure followed by one unsignaled footshock for 2 s at a strain specific intensity (129S1 and 129S6 at 0.5 mA and C57B6 at 0.75 mA). Mice were removed from the conditioning chambers 30 s after the footshock. In context B, mice received context exposure for 3 min and 32 s, comparable to the time spent in context A with no unsignaled footshock. Mice were tested for their fear to contexts A and B on day 10 using 3 min and 30 s exposure to each context in the absence of a footshock. Roughly 24 h later, mice were tested in the different context, context C, using 5 min of context exposure to test for increases in basal anxiety.

##### Tone protocols

Mice were fear conditioned to a tone in context A using three training sessions, one per day for 3 days. During training, mice were exposed to context A for 3 min followed by three tone-shock presentations in which a 30 s tone (75 dB, 2.8 kHz) co-terminated with a 2 s footshock, with 30 s between tones. Mice remained in the conditioning chamber for 30 s following the last tone-shock pairing. Mouse strains 129S6 and 129S1 received footshocks at 0.5 mA and strain C57B6 at 0.75 mA. Seventy-two hours later, mice were tested to their fear of the tone in context C using 1 min context exposure followed by three 30 s tone-alone presentations spaced 30 s apart. Mice were removed from the chambers 30 s after the last tone presentation. For tone extinction experiments, mice were divided into two groups following training: extinction and no extinction. Twenty-four hours after training, mice in the extinction group received 2 days of extinction training in context B with each day consisting of 2 min of context exposure followed by 30 30-s tone-alone presentations spaced 30 s apart. Mice were removed from the conditioning chambers 30 s after the last tone presentation. In lieu of extinction, mice in the no extinction group were placed in context C for 32 min per day for 2 days with no tone presentations. Twenty-four hours after extinction training, no extinction and extinction mice were tested for their fear of the tone using the testing protocol described above. To study acquisition of fear to the tone, fear of the tone in the no extinction group during testing was observed.

## Results

### Similar acquisition and consolidation of fear memories in the 129S6 and C57B6 strains

To assess whether 129S6 mice showed deficits in fear acquisition and/or consolidation, 129S6 mice were compared to the commonly used C57B6 mice using Pavlovian fear conditioning to a context and tone.

**Figure 1:**
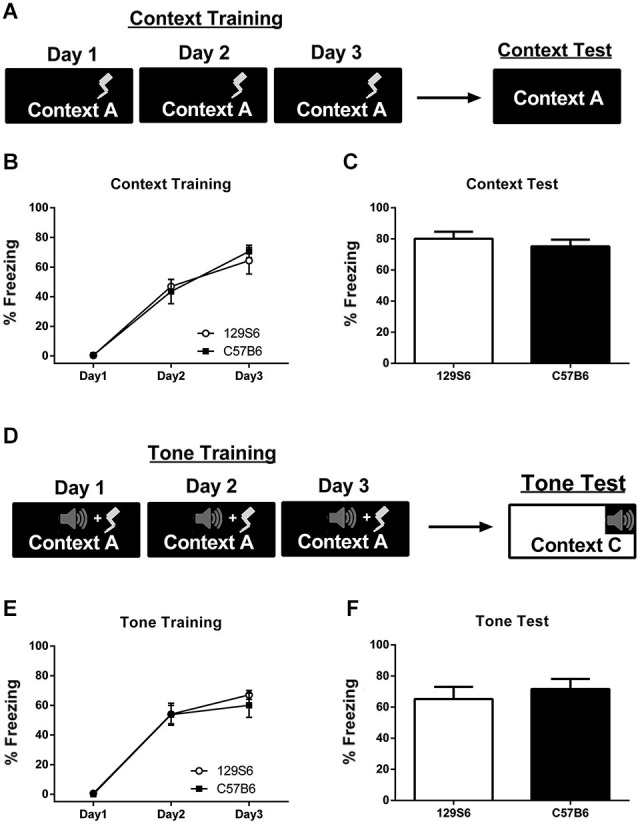
**Inbred mouse strains, 129S6 and C57B6, have comparable levels of fear acquisition and consolidation**. **(A)** Schematic representation of context fear conditioning. Mice were trained to a context or tone using 3 unsignaled footshocks or 3 tone-shock pairings per day for 3 days. Acquisition of fear across training is represented as the average percent freezing to the context or tone prior to shock for each day. Mice were tested 24 h later. **(B)** The 129S6 mice (*n* = 7) showed a significant increase in freezing across context training days comparable to that seen in C57B6 mice (*n* = 8). **(C)** During testing, both 129S6 and C57B6 mice show high levels of freezing to the context, not significantly different from each other. **(D)** Schematic represention of tone fear conditioning. **(E,F)** Similar results were obtained when 129S6 and C57B6 mice (*n* = 4 and 4 respectively) were conditioned to a tone and tested 72 h later. Data are presented as mean ± SEM. * *p* < 0.05.

Mice were fear conditioned to a context using 3 min of context exposure followed by 3 unsignaled footshocks per day for 3 days (Figure 1A). Acquisition of fear across training days was analyzed as the average percent freezing over the first 3 min of context exposure for each day (Figure 1B). Analysis of fear acquisition across training days using a repeated measures ANOVA showed a significant effect of training (*F*_(2, 26)_ = 103.19, *p* < 0.0001), but no significant difference between 129S6 and C57B6 strains (*F*_(1, 13)_ = 0.008, *p* = 0.9321). Twenty-four hours after fear conditioning, mice were tested for fear to the trained context using 5 min of context exposure (Figure 1C). Analysis of context testing using an unpaired *t*-test showed no significant difference in percent time freezing between strains (*p* = 0.966) with 129S6 and C57B6 mice freezing at an average of 80% and 75% respectively. A separate group of mice were fear conditioned to a tone using 3 min of context exposure followed by three tone-shock pairings per day for 3 days (Figure 1D). Average freezing to the tone was calculated per day. Analysis of fear acquisition across tone training days using a repeated measures ANOVA showed no significant difference between strains (*F*_(1, 6)_ = 0.167, *p* = 0.689) but a significant effect of training (*F*_(2,12)_ = 119.35, *p* < 0.001) (Figure 1E). Seventy-two hours later mice were tested for their fear to a tone in a different context using five tone-alone presentations (Figure 1F). During testing, there was no significant difference between strains (*p* = 0.6512) with 129S6 mice showing an average freezing level of 69% compared with 75% in C57B6 mice. These results demonstrate that the 129S6 mice exhibit normal, and adaptive, fear learning to a context and tone.

### Mouse strain 129S6 shows significant deficits in cued and contextual fear extinction compared to C57B6 mice

**Figure 2:**
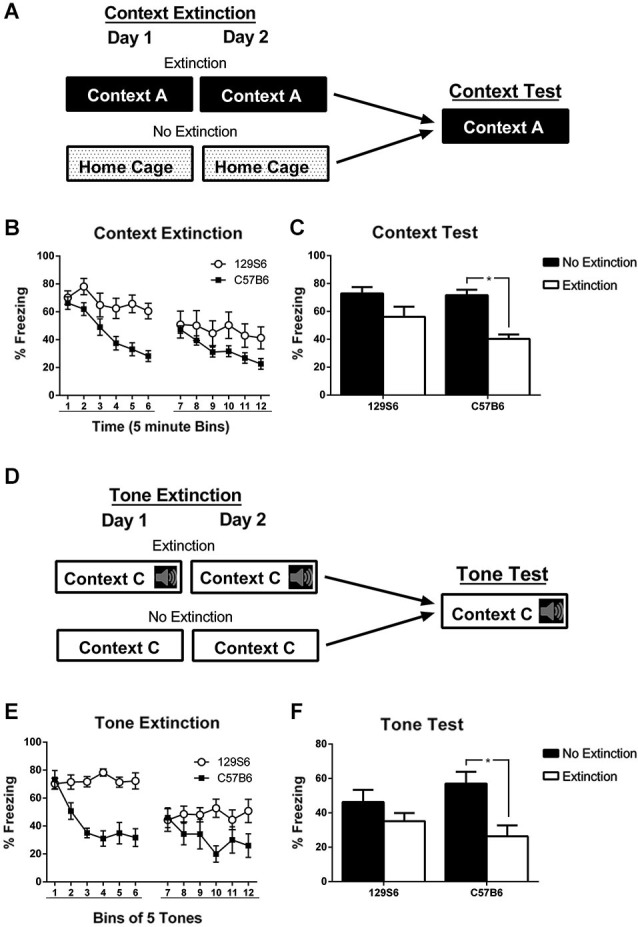
**Inbred mouse strain 129S6 shows deficits in context and tone extinction**. **(A)** Schematic representation of context extinction. Mice were trained to a context or tone using 3 tone-shock or unsignaled footshock presentations per day for 3 days. Subsequently, mice were split into extinction and no extinction groups. Mice that were trained to a context were extinguished to the training context using 60 min of context exposure split across 2 days. Mice that were trained to fear a tone were extinguished with 60 tone-alone presentations split across 2 days. Extinction data is binned in groups of 5 min or 5 tones with bins 1–6 representing day 1 of extinction and bins 7–12 representing day 2. Twenty four hours later all mice (both the extinction and no extinction groups) were exposed to the training context or the training tone in a novel context. **(B)** During context extinction, 129S6 showed high levels of freezing within extinction days 1 and 2 compared to C57B6 mice. **(C)** During testing, C57B6 mice in the extinction group (*n* = 16) showed significantly less freezing than the no extinction controls (*n* = 8), while 129S6 mice exhibited similar levels of freezing across both groups (*n* = 13 and 7 for the extinction and no extinction groups, respectively). **(D)** Schematic representation of tone extinction. **(E,F)** Similar results were observed when C57B6 mice (*n* = 7 and *n* = 8; extinction and no extinction groups, respectively) and 129S6 mice (*n* = 8 and *n* = 9; extinction and no extinction groups, respectively) were extinguished and tested with a tone. Data are represented as mean ± SEM. * *p* < 0.05.

**Figure 3:**
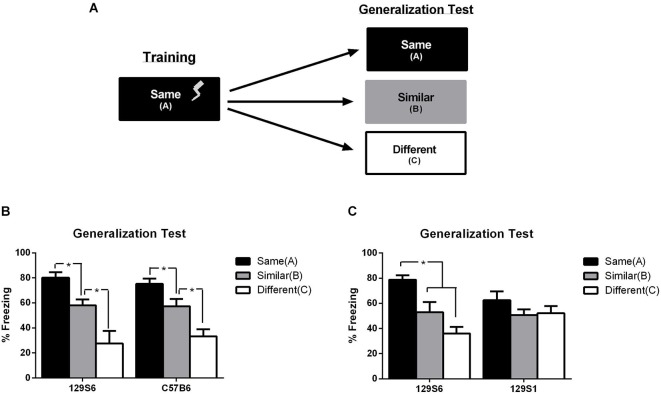
**Inbred mouse Strain 129S6, but not 129S1, has normal context generalization**. **(A)** Schematic representation of experiment. Mice were trained to a context using 3 unsignaled footshock presentations per day for 3 days. During generalization testing, mice were tested for fear in either the same training context, a similar context, or a different context. Data are represented as the average percent freezing in each context. **(B)** When tested, both 129S6 and C57B6 mice displayed high levels of freezing in the same context (C57B6 *n* = 8, 129S6 *n* = 7), intermediate levels of freezing in the similar context (C57B6 *n* = 8, 129S6 *n* = 6), and low levels of freezing in the different context (C57B6 *n* = 8, 129S6 *n* = 7). **(C)** In a separate experiment, fear generalization in the 129S6 and 129S1 substrains were compared. Consistent with previous reports, the 129S1 mice exhibited similar levels of freezing regardless of the context (same, *n* = 8; similar, *n* = 8; different, *n* = 8). In contrast, and consistent with our previous experiment, the 129S6 exhibited graded levels of freezing in the three contexts (same, *n* = 8; similar, *n* = 8; different, *n* = 8). Data are represented as mean ± SEM. * *p* < 0.05.

Our results demonstrate that the 129S6 strain exhibit normal acquisition and consolidation compared to C57B6, which is similar to results obtained with the 129S1 strain (Hefner et al., 2008). To determine whether 129S6 mice have deficits in fear extinction, 129S6 and C57B6 mice were conditioned to a context or a tone then extinguished by repeated CS exposures. Mice were first fear conditioned to a context using 3 min of context exposure followed by 3 unsignaled footshocks per day for 3 days, same as the context fear conditioning described above. Twenty-four hours later, mice were tested for fear generalization (see below and Figure 3A). The following day mice were counter balanced and divided into extinction and no extinction groups. Mice in the extinction group received 30 min of context exposure per day for 2 days while mice in the no extinction group remained in their home cage (Figure 2A).

To assess fear extinction learning between strains, freezing responses to the trained context were analyzed in 5 min time bins with bins 1–6 representing extinction day 1 and bins 7–12 representing extinction day 2 (Figure 2B). Within session fear extinction was analyzed using a repeated measures ANOVA which revealed a significant effect of extinction training in 129S6 mice on day 1 (*F*_(5, 50)_ = 4.037, *p* = 0.0032), but not on extinction day 2 (*F*_(5, 50)_ = 1.871, *p* = 0.1129). In comparison, analysis of within session fear extinction in C57B6 mice revealed a significant effect of extinction training on extinction day 1 (*F*_(5, 60)_ = 24.970, *p* < 0.0001) and extinction day 2 (*F*_(5, 60)_ = 7.334, *p* < 0.0001). Additionally, analysis of within session extinction using a repeated measures ANOVA also revealed a significant effect of strain on both extinction day 1 (*F*_(1, 27)_ = 7.334, *p* = 0.0116) and day 2 (*F*_(1, 27)_ = 25.907, *p* < 0.0001). Twenty-four hours after extinction, all mice were tested for their fear to the trained context. Analysis of context freezing using a two-way ANOVA followed by *post hoc* analysis with a planned unpaired *t*-test found a significant effect of extinction training (*F*_(1,42)_ = 18.772, *p* < 0.0001), which was significant in C57B6 mice (*p* < 0.0001), but not 129S6 mice (*p* = 0.0991) (Figure 2C).

To determine whether 129S6 mice exhibit deficits in tone fear extinction, mice were trained to a tone using three tone shock pairings per day for 3 days, as described above. Twenty-four hours later, mice were split into extinction and no extinction groups (Figure 2D). Mice in the extinction group were extinguished in a novel context, Context C, using 30 tone alone presentations per day for 2 days. Mice in the no extinction group were placed in the novel context, without tone presentations, for an equivalent length of time. Extinction training was plotted similarly to context extinction with bins of five tones and bins 1–6 representing extinction day 1 and 7–12 representing extinction day 2 (Figure 2E). Analysis of within-session fear extinction using repeated measures ANOVA revealed a no significant effect of extinction training in 129S6 mice on extinction day 1 (*F*_(5,25)_ = 0.704, *p* = 0.6239) or extinction day 2 (*F*_(5,25)_ = 0.866, *p* = 0.5138). In comparison, analysis of within-session extinction in C57B6 mice found a significant effect of extinction training on both extinction day 1 (*F*_(5,20)_ = 11.284, *p* < 0.0001) and extinction day 2 (*F*_(5,20)_ = 3.734, *p* < 0.0001). Analysis of within-session fear extinction between strains using a repeated measures ANOVA revealed a significant effect of strain on extinction day 1 (*F*_(1, 30)_ = 37.894, *p* < 0.0001) and day 2 (*F*_(1, 30)_ = 7.761, *p* = 0.0092). Twenty-four hours later mice were tested for their fear to the trained tone (Figure 2F). Analysis of tone testing using a two-way ANOVA found a significant effect of extinction training (*F*_(1, 30)_ = 10.580, *p* = 0.0030). Further analysis using a planned unpaired *t*-test revealed that the effect of extinction was limited to the C57B6 mice (*p* = 0.0064) with no significant difference between the extinction and no extinction groups in the 129S6 mice (*p* = 0.2221). This data indicates that, like 129S1 mice, 129S6 mice have significant deficits in fear extinction to a context as well as a tone. This is consistent with the previously published data suggesting extinction deficits in various genetically similar 129 strains (Hefner et al., 2008; Camp et al., 2009).

### Mouse strain 129S6 shows comparable/normal levels of fear generalization as C57B6 mice

Based on the similarities in fear conditioning and extinction phenotypes between 129S1 and 129S6, we hypothesized that 129S6 mice would also show an overgeneralization of fear from a trained context to a novel and similar context, as previously seen in 129S1 mice (Camp et al., 2012). To test for fear generalization, mice that were trained to context “A”, using the context training protocol described above were tested for generalization of fear to an untrained context. Twenty-four hours after training, animals were tested for their fear to either the trained context A, a similar context B, or a completely different context C (Figure 3A). See Section “Materials and Methods” for further details pertaining to the similarities and differences between contexts. In mice considered to generalize normally, context B should produce some level of generalized fear due to its similarities to context A, but this fear, represented as percent time freezing, would be expected to be significantly lower than that seen in the trained context. Due to the substantially different nature of context C, mice considered to generalize normally should show significantly lower levels of freezing compared to that seen in the trained context, and in many cases, significantly lower levels of freezing than that seen to the similar context. Fear was analyzed as an average percent freezing in each context and compared using a two-way ANOVA with strain and context as factors. Analysis of context generalization showed no interaction between strain and context (*F*_(1, 43)_ = 0.075, *p* = 0.7855), but a significant effect of context (*F*_(2, 86)_ = 22.444, *p* < 0.0001). Using an unpaired *t*-test between contexts it was found that both C57B6 and 129S6 froze significantly more in the same context, at 80% in 129S6 mice and 75% in C57B6 mice, than in either the similar context (129S6:*p* = 0.0481, C57B6:*p* = 0.0271) or different context (129S6:*p* = 0.0027, C57B6:*p* < 0.0001). Furthermore, both C57B6 and 129S6 froze at a significantly higher level in the similar context, at 58% in 129S6 mice and 57% in C57B6 mice, than the different context (129S6:*p* = 0.0156, C57B6:*p* = 0.0106). Both 129S6 and C57B6 mice also showed low levels of freezing in the different context at 28% and 33% respectively (Figure 3B). This data indicates that 129S6 mice show similar levels of fear generalization as C57B6 mice, suggesting normal levels of fear generalization. While these data clearly suggest normal levels of fear acquisition in 129S6 mice, this does not match previously published data showing a strong overgeneralization phenotype in the genetically similar 129S1 substrain (Camp et al., 2012).

To better understand the potential differences in 129S6 and 129S1 mice in fear generalization and to rule out a difference in generalization parameters between current and previously reported studies in the mouse phenotypes observed, 129S1 mice were compared to 129S6 mice in the fear generalization protocol previously described. Analysis of fear generalization between 129S1 and 129S6 mice using a two-way ANOVA showed a significant interaction of strain and context (*F*_(2, 170)_ = 8.404, *p* = 0.0003) as well as context (*F*_(2, 170)_ = 23.580, *p* < 0.0001; Figure 3C). *Post hoc* analysis using planned unpaired *t*-tests showed significantly higher levels of freezing of 129S6 mice in the same context at 79% compared with the similar context at 53% (*p* = 0.015) and different context at 36% (*p* < 0.001). Conversely, there was no significant difference between 129S1 freezing in the same context at 63% compared to the similar (*p* = 0.173) and different (*p* = 0.842) contexts. Taken together, these results support a strong fear overgeneralization phenotype in 129S1 mice which is absent in 129S6 mice, despite the similarities between these two substrains in persistent/extinction-resistant fear.

### Unlike 129S1 mice, 129S6 can be trained to discriminate between similar contexts

**Figure 4:**
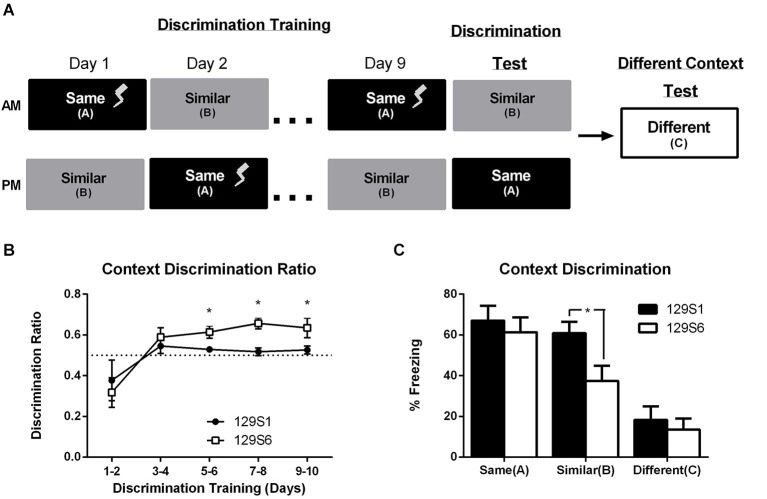
**Inbred mouse strain 129S1, but not 129S6, fail to context discriminate independent of an increase in basal anxiety**. **(A)** Mice were trained to context discriminate using a daily exposure to context A and context B in which only the exposure to context A was paired with a single unsignaled footshock on days 1 through 9. On day 10 mice were returned to context A and context B and the shock was omitted. Mice were tested for fear to a different context (context C) 1 day later. **(B)** Context Discrimination across training days is represented as a discrimination ratio of freezing in context A divided by freezing in context B with 0.5 representing no discrimination (dotted line). Ratios are averaged every 2 days to counterbalance for order of context exposure. During context discrimination training, 129S6 mice show significant context discrimination on day 5–10, while 129S1 mice failed to exhibit significant context discrimination. * *p* < 0.05 compared to chance (0.5 ratio; dotted line). **(C)** On day 10, exposure to context A produced similar levels of freezing in both 129S6 and 129S1 substrains. However, in context B, 129S1 mice exhibited significantly more freezing compared to 129S6 mice. Conversely, both substrains exhibited similarly low levels of freezing when exposed to a novel context (context C). Data are presented as mean ± SEM; * *p* < 0.05 *n* = 16 for 129S1 and 129S6.

Our results demonstrate that unlike the 129S1 substrain, the 129S6 mice exhibit an intact generalization of fear gradient. We hypothesized that the differences in 129S6 and 129S1 substrain fear generalization might be due to an increase in anxiety-like behavior in the 129S1 strain. To further investigate the putative substrain differences and to better understand whether the overgeneralization in 129S1 mice represents an inability to cognitively discriminate between two similar contexts or an overall increase in anxiety-related behavior independent of context, we subjected both substrains to a context discrimination paradigm. During context discrimination training, mice were exposed to two contexts, contexts A and B, each day for 10 days. In context A, mice received 3 min of context exposure followed by one unsignaled footshock and were then removed 30 s later. In context B, mice did not receive a footshock and were instead allowed an equivalent period of time (3.5 min) of context exposure and removed from the chamber. The order in which mice received contexts A and B was alternated each day to control for order and time of day effects (Figure 4A). On day 10, discrimination test, mice were placed back in contexts A and B in the absence of a footshock. Twenty-four hours after training, mice were placed in a novel context, context C. Fear learning to each context was analyzed throughout training by comparing the percent freezing in the first 3 min of exposure to each context per day across days. Analysis using a repeated measures ANOVA revealed a significant effect of training day in context A (*F*_(4, 120)_ = 87.133, *p* < 0.0001) and no significant difference between strains (*F*_(1, 30)_ = 0.8795, *p* = 0.6194) (data not shown). Discrimination ratios were calculated for each day of training as the percent freezing in context A divided by the sum of freezing in contexts A and B. A discrimination ratio of 0.5 represents a lack of context discrimination. To control for the effect of time of day on freezing to each context, discrimination ratios are represented as an average of 2 days (Figure 4B). When compared to chance (a discrimination ratio of 0.5) 129S6 mice were able to discriminate between the two similar contexts by the fifth/sixth day of training (*p* = 0.0079), whereas the discrimination ratio for mice in the 129S1 strain never exceeded chance (*p* = 0.2344 on days 9/10) (Figure 4B). These data, similar to the generalization data presented above, suggest that 129S1 mice may be prone to overgeneralization of fear in context B. Both 129S6 and 129S1 mice show an average freezing level of over 60% in context A with no significant difference between the strains during discrimination testing (Figure 4C). However, while 129S6 mice showed a significantly lower level of freezing in context B, compared to context A (*p* = 0.0377), 129S1 mice showed high levels of freezing in context B that was indistinguishable from that seen in context A (*p* = 0.5192) and significantly higher than 129S6 mice (*p* = 0.026). If the inability to actively discriminate between context A and context B in the 129S1 strain is due to an enhanced anxiety state, one would predict that the 129S1 mice would exhibit high levels of freezing in a novel context compared to 129S6. However, when mice were exposed to a novel context (context C) both 129S1 and 129S6 mice exhibited similarly low levels of freezing (*p* = 0.5864). This data suggests that 129S1 mice, unlike 129S6 mice, overgeneralize their fear of a trained context to a similar context and that this generalization is likely due to an inability to discriminate between the two contexts vs. an increase in anxiety-like behavior due to fear conditioning.

## Discussion

Through a series of fear related studies, we were able to segregate different forms of maladaptive fear in two genetically similar substrains of inbred mice. While 129S6 mice show strong deficits in fear extinction, similar to the previous published 129S1 mice; 129S1, but not 129S6 mice, show substantial fear over generalization and a lack of context discrimination.

Our studies found that 129S6 mice exhibit severe deficits in fear extinction in spite of normal learning and consolidation of fear memories. While 129S6 and C57B6 mice froze at comparable levels throughout training and at the start of extinction training, 129S6 mice continued to freeze at high levels throughout the extinction training sessions. In addition, unlike the C57B6 mice, the 129S6 mice maintained high levels of freezing when tested on subsequent test days, exhibiting similar levels of freezing to mice in the no extinction group. These findings are consistent with previous experiments that have described similar deficits in other 129 substrains (Hefner et al., 2008; Camp et al., 2009).

Despite 129S6 mice exhibiting clear aberrant fear processing in terms of extinction learning, data from our experiments demonstrates normal levels of fear generalization in the 129S6 substrain. Both 129S6 and C57B6 mice showed high levels of fear to the trained context, illustrating normal fear learning, and significantly lower levels of fear to both a similar and completely novel context, suggesting a normal generalization of fear gradiant. However, when we directly compared the 129S6 and 129S1 substrains, 129S1 mice exhibited overgeneralization of fear with high levels of freezing in both the trained and similar, as well as in the completely novel context. These data suggest an inability of 129S1 mice to distinguish between the contexts or an enhanced state of anxiety induced by fear conditioning. However, our studies comparing context discrimination between the 129S1 and 129S6 mice found a lack of discrimination in the 129S1 mice which was independent from an increase in overall anxiety. While 129S6 mice exhibited significantly higher levels of fear to the trained context vs. the similar context, illustrated by a discrimination ratio of >0.5, 129S1 mice showed a similar fear response in both contexts resulting in a discrimination ratio of roughly 0.5. However, 129S1 and 129S6 exhibited similarly low levels of freezing to a completely different context. Taken together with the fear generalization data, this deficit in context discrimination suggests that the differences in 129S1 mice regarding overgeneralization is likely due to an inability to cognitively separate the two contexts. While unexpected, these discrepancies between two genetically similar substrains suggests clear differences in the mechanisms underlying fear overgeneralization and persistent fears that may be tied to the subtle differences in genotypes.

In recent years a great deal has been uncovered regarding the neurobiology that underlies fear learning and extinction (Myers and Davis, 2007; Milad and Quirk, 2012; Orsini and Maren, 2012). Previous studies have linked deficits in fear extinction to a context or cue to various brain structures including the hippocampus (Corcoran et al., 2005), prefrontal cortex (PFC; Milad and Quirk, 2002), and amygdala (Likhtik et al., 2008). In addition a number of cellular changes have been implicated, including presynaptic changes (Stork et al., 2002; Tsvetkov et al., 2002) and alterations in protein synthesis (Hernandez and Abel, 2008). While the mechanism underlying the extinction deficits in 129S6 substrain has yet to be determined, previous reports comparing 129S1 mice and C57B6 mice have found altered neuronal activity in the infralimbic cortex of the PFC, basolateral amygdala, and the central amygdala in 129S1 mice associated with fear extinction (Hefner et al., 2008). These changes in neuronal activity were measured as alterations in the expression of immediate early genes (IEG) c-Fos and Zif268 and implicate the PFC and amygdala function in the extinction deficits seen in 129S1 mice, and perhaps other 129 substrains, such as 129S6. These results were further supported by studies suggesting that a zinc restricted diet improved fear extinction learning in 129S1 mice and normalized the expression of IEGs in the amygdala and PFC compared to C57B6 mice (Whittle et al., 2010). While it is possible that these changes in neuronal activity in the PFC and amygdala in129S1 mice may also be responsible for the generalization and context discrimination deficits described in this substrain, the presence of extinction deficits, but not overgeneralization in the 129S6 substrain suggests a disassociation between these two forms of maladaptive fear.

In this case, deficits in the cognitive ability to learn and distinguish two contexts likely involves structures known to be involved in memory formation. In particular, the ability to encode fine details regarding a pattern or context and distinguish this information from other patterns or contexts has been linked to the dentate gyrus of the hippocampal formation (Gilbert et al., 2001; Leutgeb et al., 2007; McHugh et al., 2007) and more specifically, the adult-born granule cells in the dentate gyrus of the hippocampal formation (Clelland et al., 2009; Sahay et al., 2011; Nakashiba et al., 2012; Tronel et al., 2012). For example, several studies that examined context discrimination have found that ablation of adult-born neurons prior to learning prevented discrimination of similar contexts (Clelland et al., 2009; Sahay et al., 2011; Nakashiba et al., 2012; Tronel et al., 2012). Conversely, increasing adult neurogenesis resulted in enhanced context discrimination (Sahay et al., 2011). Interestingly, previous studies of various mouse strains have noted significant differences in basal rates of adult neurogenesis in dentate gyrus, including lower levels of neurogenesis in 129S1 mice compared to C57B6 mice (Kempermann et al., 1997; Kempermann and Gage, 2002). At present it remains unknown to what extent these differences in basal levels of neurogenesis might alter behavioral output measures in general, or whether these differences in adult neurogenesis might account for differences in context discrimination, as seen in the 129S1 and 129S6 mouse strains. In addition, it remains unknown whether strain dependent variations in survival and functional integration of adult born neurons may also play a role in learning and memory. Careful examination of strain dependent behavioral differences, similar to the current study, in conjunction with detailed cellular analysis could be used in the future to further explore the putative link between adult neurogenesis and cognitive function.

In the current study we describe significant substrain differences in aberrant generalization of fear and context discrimination but not extinction of conditioned fear. Functionally, similarities between 129S6 and 129S1 in extinction deficits may be linked to the similarities in substrain genetics absent in C57B6 mice, while differences in 129S6 and 129S1 mice in fear generalization and context discrimination may be mediated by differences in genetics between the two substrains. These similarities and differences in 129S6 and 129S1 mice may highlight important differences between two types of maladaptive fear: that associated with an inability to extinguish to a CS and that associated with an overgeneralization of fear to an US. This dissociation between maladaptive fears suggests different mechanisms and underlying genetics that give rise to these two forms of maladaptive fear. Identification of the genes and cellular mechanisms involved in the distinct forms of maladaptive fear may lead to a better understanding of their roles in trauma and anxiety-related disorders and their treatments. While the use of inbred mouse strains to model anxiety-related and trauma-related disorders is limited due to the genetic homogeny and limited behavioral variability of inbred mouse strains compared to the heterogeneity of the human population, this homogeneity can be useful in the identification of genes and mechanisms mediating specific types of behaviors (Sankoorikal et al., 2006). Though unable to directly model the psychiatric disorder itself, discoveries such as these can inform studies aimed at understanding specific components leading to various pathological behaviors and conditions (Mahler et al., 1998; Lifsted et al., 1998; Qi et al., 2005; Sankoorikal et al., 2006). In this way, 129S1 and 129S6 mice can function as key tools for studying the mechanisms underlying these maladaptive fears individually. Given the presumed genetic similarities of the 129S1 and 129S6 substrains (Simpson et al., 1997) and the divergent forms of maladaptive fear described here, we suggest that further comparison of the two substrains may reveal valuable insights into psychiatric disease states thought to be related to maladaptive fear learning and or memory.

## Conflict of interest statement

The authors declare that the research was conducted in the absence of any commercial or financial relationships that could be construed as a potential conflict of interest.

## References

[B1] Balogh S. A. Wehner J. M. (2003). Inbred mouse strain differences in the establishment of long-term fear memory. Behav. Brain Res. 140, 97–106 10.1016/s0166-4328(02)00279-6 12644283

[B2] Bolivar V. J. Pooler O. Flaherty L. (2001). Inbred strain variation in contextual and cued fear conditioning behavior. Mamm. Genome 12, 651–656 10.1007/s003350020039 11471061

[B3] Bothe G. W. Bolivar V. J. Vedder M. J. Geistfeld J. G. (2004). Genetic and behavioral differences among five inbred mouse strains commonly used in the production of transgenic and knockout mice. Genes Brain Behav. 3, 149–157 10.1111/j.1601-183x.2004.00064.x 15140010

[B4] Bouton M. E. King D. A. (1983). Contextual control of the extinction of conditioned fear: tests for the associative value of the context. J. Exp. Psychol. Anim. Behav. Process. 9, 248–265 10.1037//0097-7403.9.3.248 6886630

[B5] Bracha H. S. (2006). Human brain evolution and the “Neuroevolutionary Time-depth Principle:” implications for the reclassification of fear-circuitry-related traits in DSM-V and for studying resilience to warzone-related posttraumatic stress disorder. Prog. Neuropsychopharmacol. Biol. Psychiatry 30, 827–853 10.1016/j.pnpbp.2006.01.008 16563589 PMC7130737

[B6] Camp M. C. Macpherson K. P. Lederle L. Graybeal C. Gaburro S. Debrouse L. M. (2012). Genetic strain differences in learned fear inhibition associated with variation in neuroendocrine, autonomic and amygdala dendritic phenotypes. Neuropsychopharmacology 37, 1534–1547 10.1038/npp.2011.340 22334122 PMC3327858

[B7] Camp M. Norcross M. Whittle N. Feyder M. D’Hanis W. Yilmazer-Hanke D. (2009). Impaired Pavlovian fear extinction is a common phenotype across genetic lineages of the 129 inbred mouse strain. Genes Brain Behav. 8, 744–752 10.1111/j.1601-183X.2009.00519.x 19674120 PMC2783364

[B8] Clelland C. D. Choi M. Romberg C. Clemenson G. D. Jr. Fragniere A. Tyers P. (2009). A functional role for adult hippocampal neurogenesis in spatial pattern separation. Science 325, 210–213 10.1126/science.1173215 19590004 PMC2997634

[B9] Corcoran K. A. Desmond T. J. Frey K. A. Maren S. (2005). Hippocampal inactivation disrupts the acquisition and contextual encoding of fear extinction. J. Neurosci. 25, 8978–8987 10.1523/jneurosci.2246-05.2005 16192388 PMC6725608

[B10] Crawley J. N. Belknap J. K. Collins A. Crabbe J. C. Frankel W. Henderson N. (1997). Behavioral phenotypes of inbred mouse strains: implications and recommendations for molecular studies. Psychopharmacology (Berl) 132, 107–124 10.1007/s002130050327 9266608

[B11] Gilbert P. E. Kesner R. P. Lee I. (2001). Dissociating hippocampal subregions: double dissociation between dentate gyrus and CA1. Hippocampus 11, 626–636 10.1002/hipo.1077 11811656

[B12] Hefner K. Whittle N. Juhasz J. Norcross M. Karlsson R. M. Saksida L. M. (2008). Impaired fear extinction learning and cortico-amygdala circuit abnormalities in a common genetic mouse strain. J. Neurosci. 28, 8074–8085 10.1523/JNEUROSCI.4904-07.2008 18685032 PMC2547848

[B13] Hernandez P. J. Abel T. (2008). The role of protein synthesis in memory consolidation: progress amid decades of debate. Neurobiol. Learn. Mem. 89, 293–311 10.1016/j.nlm.2007.09.010 18053752 PMC2745628

[B14] Holmes A. Wrenn C. C. Harris A. P. Thayer K. E. Crawley J. N. (2002). Behavioral profiles of inbred strains on novel olfactory, spatial and emotional tests for reference memory in mice. Genes Brain Behav. 1, 55–69 10.1046/j.1601-1848.2001.00005.x 12886950

[B15] Kempermann G. Gage F. H. (2002). Genetic influence on phenotypic differentiation in adult hippocampal neurogenesis. Brain Res. Dev. Brain Res. 134, 1–12 10.1016/s0165-3806(01)00224-3 11947932

[B16] Kempermann G. Kuhn H. G. Gage F. H. (1997). Genetic influence on neurogenesis in the dentate gyrus of adult mice. Proc. Natl. Acad. Sci. U S A 94, 10409–10414 10.1073/pnas.94.19.10409 9294224 PMC23376

[B17] Leutgeb J. K. Leutgeb S. Moser M. B. Moser E. I. (2007). Pattern separation in the dentate gyrus and CA3 of the hippocampus. Science 315, 961–966 10.1126/science.1135801 17303747

[B18] Lifsted T. Le Voyer T. Williams M. Muller W. Klein-Szanto A. Buetow K. H. (1998). Identification of inbred mouse strains harboring genetic modifiers of mammary tumor age of onset and metastatic progression. Int. J. Cancer 77, 640–644 10.1002/(sici)1097-0215(19980812)77:4<640::aid-ijc26>3.0.co;2-8 9679770

[B19] Likhtik E. Popa D. Apergis-Schoute J. Fidacaro G. A. Pare D. (2008). Amygdala intercalated neurons are required for expression of fear extinction. Nature 454, 642–645 10.1038/nature07167 18615014 PMC2528060

[B20] Mahler M. Bristol I. J. Leiter E. H. Workman A. E. Birkenmeier E. H. Elson C. O. (1998). Differential susceptibility of inbred mouse strains to dextran sulfate sodium-induced colitis. Am. J. Physiol. 274(3 Pt. 1), G544–G551 9530156 10.1152/ajpgi.1998.274.3.G544

[B21] Maren S. (2005). Building and burying fear memories in the brain. Neuroscientist 11, 89–99 10.1177/1073858404269232 15632281

[B22] Matynia A. Anagnostaras S. G. Wiltgen B. J. Lacuesta M. Fanselow M. S. Silva A. J. (2008). A high through-put reverse genetic screen identifies two genes involved in remote memory in mice. PLoS One 3:e2121 10.1371/journal.pone.0002121 18464936 PMC2373872

[B23] McHugh T. J. Jones M. W. Quinn J. J. Balthasar N. Coppari R. Elmquist J. K. (2007). Dentate gyrus NMDA receptors mediate rapid pattern separation in the hippocampal network. Science 317, 94–99 10.1126/science.1140263 17556551

[B25] Milad M. R. Quirk G. J. (2002). Neurons in medial prefrontal cortex signal memory for fear extinction. Nature 420, 70–74 10.1038/nature01138 12422216

[B24] Milad M. R. Quirk G. J. (2012). Fear extinction as a model for translational neuroscience: ten years of progress. Annu. Rev. Psychol. 63, 129–151 10.1146/annurev.psych.121208.131631 22129456 PMC4942586

[B26] Milad M. R. Rauch S. L. Pitman R. K. Quirk G. J. (2006). Fear extinction in rats: implications for human brain imaging and anxiety disorders. Biol. Psychol. 73, 61–71 10.1016/j.biopsycho.2006.01.008 16476517

[B27] Myers K. M. Davis M. (2007). Mechanisms of fear extinction. Mol. Psychiatry 12, 120–150 10.1038/sj.mp.4001939 17160066

[B28] Nakashiba T. Cushman J. D. Pelkey K. A. Renaudineau S. Buhl D. L. McHugh T. J. (2012). Young dentate granule cells mediate pattern separation, whereas old granule cells facilitate pattern completion. Cell 149, 188–201 10.1016/j.cell.2012.01.046 22365813 PMC3319279

[B29] Orsini C. A. Maren S. (2012). Neural and cellular mechanisms of fear and extinction memory formation. Neurosci. Biobehav. Rev. 36, 1773–1802 10.1016/j.neubiorev.2011.12.014 22230704 PMC3345303

[B30] Qi Z. Fujita H. Jin J. Davis L. S. Wang Y. Fogo A. B. (2005). Characterization of susceptibility of inbred mouse strains to diabetic nephropathy. Diabetes 54, 2628–2637 10.2337/diabetes.54.9.2628 16123351

[B31] Rauch S. L. Shin L. M. Phelps E. A. (2006). Neurocircuitry models of posttraumatic stress disorder and extinction: human neuroimaging research–past, present and future. Biol. Psychiatry 60, 376–382 10.1016/j.biopsych.2006.06.004 16919525

[B33] Rescorla R. A. (2001). Retraining of extinguished Pavlovian stimuli. J. Exp. Psychol. Anim. Behav. Process. 27, 115–124 10.1037//0097-7403.27.2.115 11296487

[B32] Rescorla R. A. Heth C. D. (1975). Reinstatement of fear to an extinguished conditioned stimulus. J. Exp. Psychol. Anim. Behav. Process. 1, 88–96 10.1037//0097-7403.1.1.88 1151290

[B34] Sahay A. Scobie K. N. Hill A. S. O’Carroll C. M. Kheirbek M. A. Burghardt N. S. (2011). Increasing adult hippocampal neurogenesis is sufficient to improve pattern separation. Nature 472, 466–470 10.1038/nature09817 21460835 PMC3084370

[B35] Sankoorikal G. M. Kaercher K. A. Boon C. J. Lee J. K. Brodkin E. S. (2006). A mouse model system for genetic analysis of sociability: C57BL/6J versus BALB/cJ inbred mouse strains. Biol. Psychiatry 59, 415–423 10.1016/j.biopsych.2005.07.026 16199013

[B36] Simpson E. M. Linder C. C. Sargent E. E. Davisson M. T. Mobraaten L. E. Sharp J. J. (1997). Genetic variation among 129 substrains and its importance for targeted mutagenesis in mice. Nat. Genet. 16, 19–27 10.1038/ng0597-19 9140391

[B37] Smith D. R. Gallagher M. Stanton M. E. (2007). Genetic background differences and nonassociative effects in mouse trace fear conditioning. Learn. Mem. 14, 597–605 10.1101/lm.614807 17823243 PMC1994077

[B38] Stork O. Ji F. Y. Obata K. (2002). Reduction of extracellular GABA in the mouse amygdala during and following confrontation with a conditioned fear stimulus. Neurosci. Lett. 327, 138–142 10.1016/s0304-3940(02)00387-7 12098654

[B39] Threadgill D. W. Yee D. Matin A. Nadeau J. H. Magnuson T. (1997). Genealogy of the 129 inbred strains: 129/SvJ is a contaminated inbred strain. Mamm. Genome 8, 390–393 10.1007/s003359900453 9166580

[B40] Tronel S. Belnoue L. Grosjean N. Revest J. M. Piazza P. V. Koehl M. (2012). Adult-born neurons are necessary for extended contextual discrimination. Hippocampus 22, 292–298 10.1002/hipo.20895 21049483

[B41] Trullas R. Skolnick P. (1993). Differences in fear motivated behaviors among inbred mouse strains. Psychopharmacology (Berl) 111, 323–331 10.1007/bf02244948 7870970

[B42] Tsvetkov E. Carlezon W. A. Benes F. M. Kandel E. R. Bolshakov V. Y. (2002). Fear conditioning occludes LTP-induced presynaptic enhancement of synaptic transmission in the cortical pathway to the lateral amygdala. Neuron 34, 289–300 10.1016/s0896-6273(02)00645-1 11970870

[B43] Whittle N. Hauschild M. Lubec G. Holmes A. Singewald N. (2010). Rescue of impaired fear extinction and normalization of cortico-amygdala circuit dysfunction in a genetic mouse model by dietary zinc restriction. J. Neurosci. 30, 13586–13596 10.1523/JNEUROSCI.0849-10.2010 20943900 PMC3149823

[B44] Wilkinson D. S. Turner J. R. Blendy J. A. Gould T. J. (2013). Genetic background influences the effects of withdrawal from chronic nicotine on learning and high-affinity nicotinic acetylcholine receptor binding in the dorsal and ventral hippocampus. Psychopharmacology (Berl) 225, 201–208 10.1007/s00213-012-2808-8 22836371 PMC3755015

